# Quercetin: A Light in the Fight Against MRSA

**DOI:** 10.1002/mbo3.70090

**Published:** 2026-03-17

**Authors:** Maria Poliana Leite Galantini, Caroline Vieira Gonçalves, Amanda Kelle Santos Novaes, Caio Oliveira Lopes de Magalhães, Igor Pereira Ribeiro Muniz, Israel Souza Ribeiro, Paulo Henrique Bispo Lima, Maria Elisa Santos Flores, Artur Reis Carvalho, Luisa Carregosa Santos, Daiana Silva Lopes, Juliano Geraldo Amaral, Robson Amaro Augusto da Silva

**Affiliations:** ^1^ Multidisciplinary Institute of Health Anísio Teixeira Campus ‐ Federal University of Bahia Vitória da Conquista Bahia Brazil; ^2^ Paulo Freire Campus – Federal University of Southern Bahia Teixeira de Freitas Bahia Brazil

**Keywords:** antimicrobial photodynamic therapy, photosensitizer, quercetin, *Staphylococcus aureus*

## Abstract

Antimicrobial photodynamic therapy (aPDT) for treating methicillin‐resistant *Staphylococcus aureus* (MRSA) infections has gained increasing attention in recent years. This study aimed to evaluate the potential of quercetin as a photosensitizer for aPDT. In vitro assays were conducted to assess the cytotoxicity and the antibacterial activity of quercetin photoactivated with blue LED light. In in vivo assays, and intradermal infection model was conducted in mice to evaluate the quercetin antimicrobial activity and cytokine production. The in vitro experiments showed low compound toxicity and antimicrobial activity accompanied by the generation of singlet oxygen. BALB/c mice infected with MRSA and treated with quercetin exhibited a reduced bacterial load in the draining lymph nodes and decreased recruitment of polymorphonuclear cells at the infection site. In response to infection, a strong interaction between the cytokines IL‐12 and TNF‐α was observed in the groups treated with quercetin. These observations support quercetin's anti‐inflammatory and antimicrobial potential in infections with resistant strains of MRSA.

## Graphical abstract

Photoactivated quercetin reduces the bacterial load of MRSA in intradermally infected mice. The control of bacterial load is associated with a modulation of the inflammatory response in animals treated with aPDT.

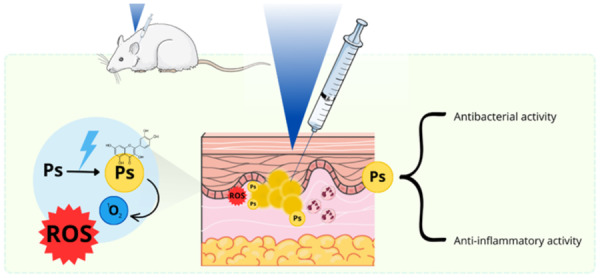

## 1 Introduction

Bacterial antimicrobial resistance has emerged as one of the significant public health threats of the 21st century and is recognized as a global health problem. With increasing resistance to multiple drugs, it is estimated that infections caused by resistant bacteria could result in up to 10 million deaths annually by 2050 (Salam et al. 2023). In this scenario, a wide range of pathogens are implicated, and resistance is high for several essential agents, including beta‐lactams and fluoroquinolones (Murray et al. 2022).

*Staphylococcus aureus* is among the primary pathogens associated with mortality due to acquired antimicrobial resistance. Methicillin‐resistant *Staphylococcus aureus* (MRSA) emerged in 1960, and currently, it is responsible for more than 100,000 deaths annually (Dweba et al. 2018; Murray et al. 2022). Microorganisms such as MRSA are challenging to control when they penetrate the skin layer. Treating these multidrug‐resistant strains represents a significant challenge for the pharmaceutical industry.

As available antibiotics become increasingly ineffective, there is a need to explore alternative therapeutic strategies. Previous studies by our group have demonstrated promising results from photodynamic treatment in the fight against MRSA (Almeida et al. 2017; dos Santos et al. 2019a, 2019b). Antimicrobial photodynamic therapy (PDT) also represents a modern alternative, minimally invasive, characterized by notably faster action against microorganisms compared with conventional antimicrobials, and, in addition, there is no evidence of resistance mechanisms developing against PDT (Fekrazad et al. 2014). Antimicrobial photodynamic therapy (aPDT) is based on the combination of a nontoxic photosensitizer (PS) and visible light of an appropriate wavelength, which in the presence of oxygen, is activated to produce reactive oxygen species (ROS) (Carrera et al. 2016). Photosensitizing agents can undergo Type I (electron transfer) and/or II (energy transfer) photochemical reactions. In the Type I reaction, electrons are transferred from the PS, resulting in the formation of free radicals. Energy is transferred to molecular oxygen for the type II reaction, generating singlet oxygen (^1^O_2_) (Ding et al. 2011). Singlet oxygen is a highly reactive species that oxidizes various biological substrates and is considered the primary mediator of photodynamic damage (Dai et al. 2012).

The choice of PS is critical for the effectiveness of PDT. An ideal PS exhibits high purity, low toxicity, chemical simplicity, stability, and the ability to generate and transfer ROS (DeRosa 2002). Several natural products have been extensively investigated as PS, including curcumin, resveratrol, and *Myrciaria cauliflora* (Almeida et al. 2017; dos Santos et al. 2019a, 2019b). Similar to these previously studied PS, quercetin is a flavonoid widely found in nature, present in various plant foods such as fruits, vegetables, teas, and grains (Deepika and Maurya 2022). It exhibits antioxidant, anti‐inflammatory, anticancer, and immunomodulatory properties (Hou et al. 2019; Yang et al. 2020; Nguyen and Bhattacharya 2022; Willian de Alencar Pereira et al. 2023). Several studies have also demonstrated that quercetin possesses antimicrobial activity against MRSA (Amin et al. 2015; Júnior et al. 2018). It can inhibit bacterial growth, interfere with bacterial biofilm formation, and even sensitize bacteria to treatment with conventional antibiotics (Abreu et al. 2016).

Given the need to better understand these phenomena, various experimental models of health and disease have been employed. In this context, the murine model of intradermal study has been used to replicate the inflammatory responses observed in humans by *S. aureus* infections (Almeida et al. 2017).

Thus, this present study aims to investigate the use of quercetin as a photosensitizer in combination with PDT in a murine model with the goal of enhancing treatment efficacy against infections caused by strains of MRSA.

## 2 Experimental Section

### 2.1 PS Preparation

Quercetin [2‐(3′,4′‐di‐hidroxi‐fenil)‐3,5,7‐tri‐hidroxi‐cromen‐4‐ona] (Val de Quimica, Brazil, lot 300402727) was used as PS. A stock solution was prepared by dissolving it in propylene glycol (LabSynth, lot 255610) and diluting it in sterile saline solution. Specifically, 0.01 g of quercetin was solubilized in 500 µL propylene and 500 µL saline, resulting in a 1:1 mixture.

### 2.2 Cell Viability and Cytotoxicity Assay

Cytotoxicity was assessed following the protocol described by Gimenes et al. (2017) using the MTT assay (3‐(4,5‐dimethylthiazol‐2‐yl)‐2,5‐diphenyltetrazolium bromide) to evaluate cell viability and cytotoxicity effects.

### 2.3 Strains, Culture Conditions, and Reagents

The ATCC 43300 MRSA strain was obtained from the Institute Multidisciplinar em Saúde collection at the University Federal da Bahia. Bacterial growth was quantified by spectrophotometry following the protocol described by Almeida et al. (2017).

### 2.4 Evaluation of In Vitro Antibacterial Activity

Different concentrations of quercetin (100, 200, 300, and 500 µg/mL) were tested, with a group containing only MRSA serving as the control. The plates were incubated in the dark for 5 min, as a pre‐irradiation period. Irradiation was performed at 450 ± 20 nm for 20 min, with an intensity of 22 mW/cm^2^ and fluence of 26.4 J/cm^2^, following the protocol described by dos Santos et al. (2019b).

### 2.5 Singlet Oxygen Production Analysis

The generation of singlet oxygen was evaluated following the protocol described by dos Santos et al. (2019b).

### 2.6 Zeta Potentials Test

The PS were incubated with bacterial suspensions of 10^8^ CFU/mL for 1 h in the dark at 37°C. Subsequently, the suspensions were centrifuged and washed three times. Zeta potentials were measured using a Malvern Zetasizer 3000HS (Zetasizer/Nanoseries), following the protocol described by Fang et al. (2016).

### 2.7 In Vivo Experimental Assays

BALB/c mice aged 6–8 weeks were used in the experiment. The animals were maintained under standard housing conditions (12‐h light/12‐h dark cycles at 22°C ± 2°C), with free access to food and water ad libitum for 12 weeks. Balb/C mice were infected intradermally in the ears with 10^8^ CFU of *S. aureus* and organized into four distinct groups: the control group (P−L−) did not receive the PS and was not exposed to the LED incidence; the P+L− group, received only the quercetin (100 µg/mL) in the same site of the infection; the P−L+ group received only blue LED light (450 nm), irradiation at the infection site and, the aPDT group (P+L+) received intradermal quercetin (100 ug/mL) in the infection site and treated with blue LED light (450 nm). For light application, the equipment was positioned 1 cm from the surface. Irradiations were carried out for 180 s (13.5 J/cm^2^), following Muniz et al. (2021). The mice were anaesthetized before each procedure with xylazine (10 mg kg^−1^) and ketamine (50 mg kg^−1^) intraperitoneally. The treatment exposure and the respective control groups occurred 24 h after infection; the animals were euthanized at 72 h postinfection. All experimental procedures were reviewed and approved by the Ethical Committee of the Federal University of Bahia (Protocol 112/2022). The animals were weighed, and the lesion size began to be measured 24 h after infection.

### 2.8 Histopathological Analysis

The ears were fixed in methacarn (70% methanol, 20% chloroform, and 10% glacial acetic acid) and processed to prepare histological slides. Histopathological analysis was performed by counting 20 image fields captured through the microscope, and a count of total inflammatory cells, mononuclear and polymorphonuclear cells was performed using the Image J software (version 1.50b, National Institute of Health, USA) Muniz et al. (2021).

### 2.9 Determination of Bacterial Load In Vivo

The retromaxillary lymph nodes were collected 72 h postinfection and macerated in a sterile Petri dish containing 500 µL of saline solution. Fifty microliters of the resulting supernatant plated on BHI medium using the pour plate method and incubated at 37°C for 24 h. Colony‐forming units (CFU) were then quantified.

### 2.10 Cytokines Levels Measurement

The cytokines TNF‐α, IL‐1β, IL‐17A, IL‐10, and IL‐12p70 in lymph node supernatants were quantified using ELISA kits, following the manufacturer's instructions (Invitrogen—ThermoFisher).

### 2.11 Statistical Analysis

Statistical differences between groups were evaluated using the Kruskal–Wallis test followed by Duns' test for non‐parametric samples. Differences were considered significant at *p* < 0.05, with a 95% confidence interval. In addition, a Bayesian network model was constructed using RStudio Version 1.0.153 software—© 2009–2017. Networks were selected based on the highest probability of based on among the analyzed factors.

## 3 Results

### 3.1 Quercetin Does Not Cause Cytotoxicity and Increases Antimicrobial Activity Under Blue LED Light

**Figure 1:**
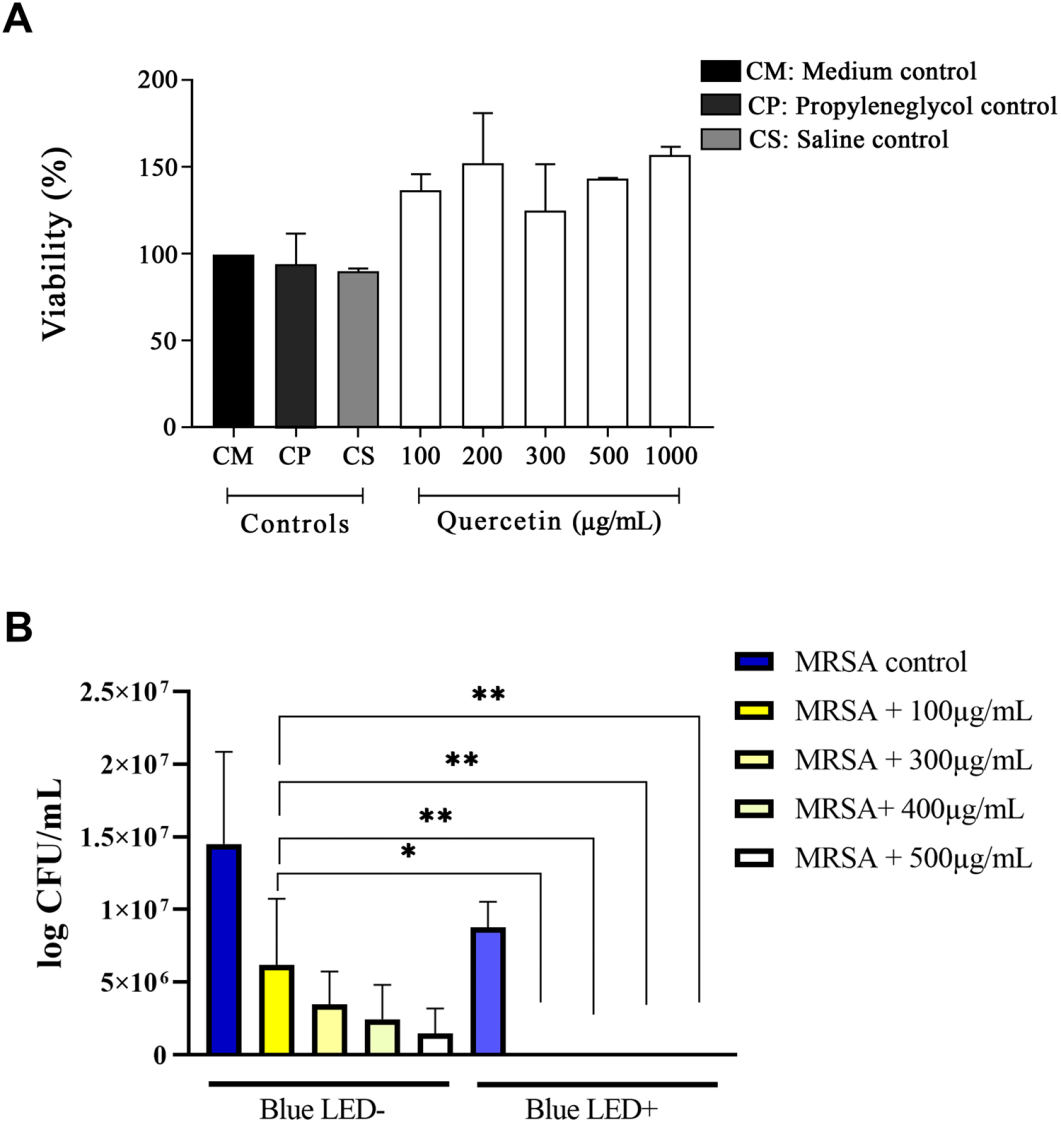
In vitro evaluation of the antibacterial potential of quercetin. Quercetin cytotoxicity was evaluated using the MTT test with HUVEC cells at concentrations of 100, 200, 300, 400, 500, and 1000 µg/mL (A). Evaluation of the in vitro photodynamic activity of quercetin against MRSA without irradiation and with blue LED light irradiation (450 nm) (B). *p < 0.05; **p < 0.001.

Cell viability was assessed using the MTT (3‐(4,5‐dimethylthiazol‐2‐yl)‐2,5‐diphenyltetrazolium bromide) assay. After 18 h of incubation, we observed that quercetin exhibited no cytotoxic effects on HUVEC cells (Figure 1A). Following the in vitro assay, photoactivation of quercetin was observed to enhance its antimicrobial activity (Figure 1B).

### 3.2 Photoactivated Quercetin Promotes Uric Acid Oxidation

**Figure 2:**
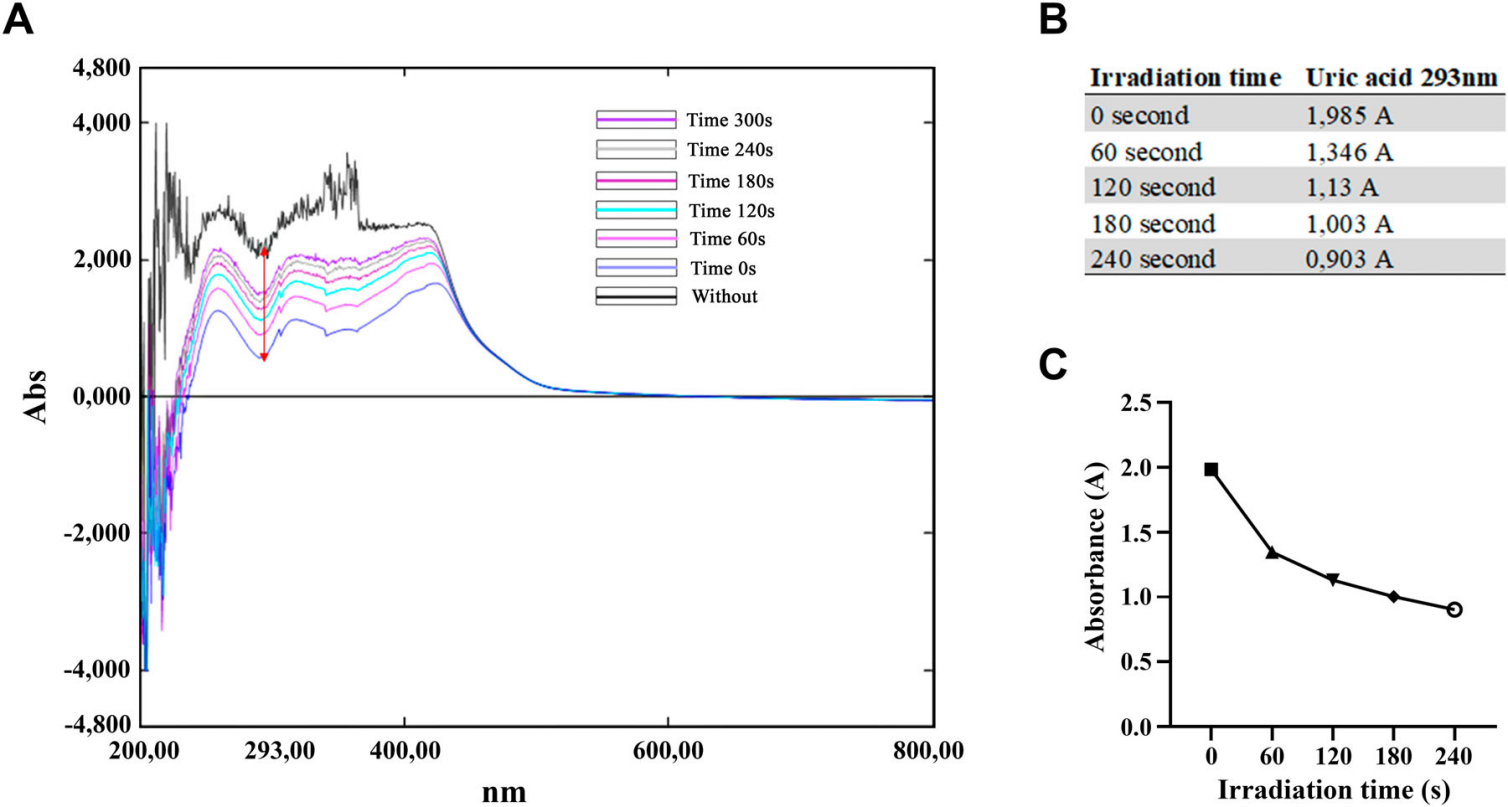
Effect of irradiation time on uric acid degradation at 293 nm with and without photoactivation. By observation of the spectral behavior, it is possible to note the decrease of uric acid band at 293 nm (A). After the photoactivation protocol, a reduction in the solution absorbance as a function of time (B) was observed. The variation of the absorbances of the two solutions as a function of time (C) was calculated. BHI, brain heart infusion; MRSA, Methicillin Resistant *Staphylococcus aureus*.

To investigate the potential antimicrobial action mechanism of photoactivated extract, an analysis was performed in the presence of uric acid (Figure 2A). The photo‐oxidation reaction of uric acid by singlet oxygen is well documented and leads to the formation of products such as triuria, allantoxaidine, oxanate ion, and CO_2_ (Santos et al, 2019b). Uric acid suppresses singlet oxygen by capturing the triplet excited state energy of quercetin. The rate of production of ROS was determined by UV‐vis spectrophotometry, monitoring the decay e kinetics of uric acid absorbance at 293 nm. It was detected that quercetin has singlet oxygen production as a mechanism of action, as shown in Figure 2C.

### 3.3 Quercetin Can Bind to the Cell Wall to Exert Its Toxic Effects

To investigate the mechanism of quercetin's antimicrobial activity, specifically its potential effects on bacterial cell membranes or DNA, zeta potential measurements were performed. Zeta potentials of MRSA, with or without the PS, were measured after washing the bacteria three times. Quercetin at a concentration of 100ug/mL, which demonstrated the strongest effects in aPDT, was selected as a test model. The vehicle exhibits negligible effects on the zeta potentials of the bacterial cell walls (−31.76 ± 0.80), whereas the negative charge density of MRSA decreased in the presence of quercetin (−36.2 ± 1.69), suggesting binding of the PS surface with the bacterial cell walls.

### 3.4 PDT Using Quercetin Reduces Bacterial Load in Draining Lymph Nodes

**Figure 3:**
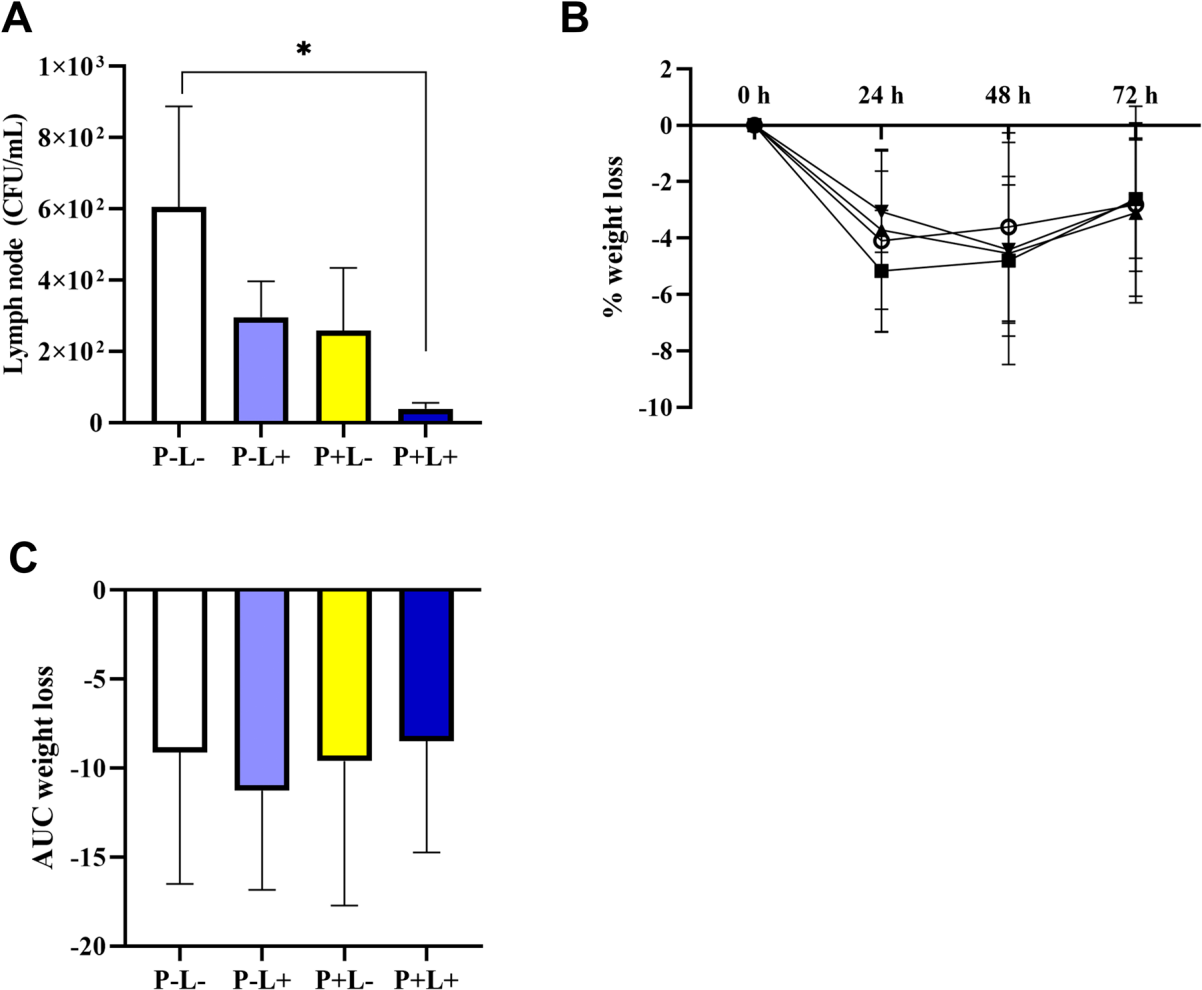
In vivo evaluation of the antibacterial potential of quercetin. (A) Bacterial load assessed in the draining lymph node with CFU quantification. (B) Animal body weight was monitored over time, showing an initial rapid loss followed by recovery, with no significant differences between groups. (C) AUC of weight loss over time. BHI, brain heart infusion; CFU, colony forming unit; AUC, area under the curve. *p < 0.05.

In the cultures of draining lymph nodes, a reduction in the number of CFU was observed in the group treated with both quercetin and light (P+L+) compared with the group exposed to light alone (P−L+). The decrease in bacterial load was statistically significant between the P+L+ group and the P−L− group at 72 h postinfection (Figure 3A). Although the P−L+ group exhibited greater weight loss after 24 h after infection, no significant differences were observed between groups (Figure 3B,C) (*p < 0.05).

### 3.5 PDT Reduces Neutrophil Recruitment to the Inflammatory Site

**Figure 4:**
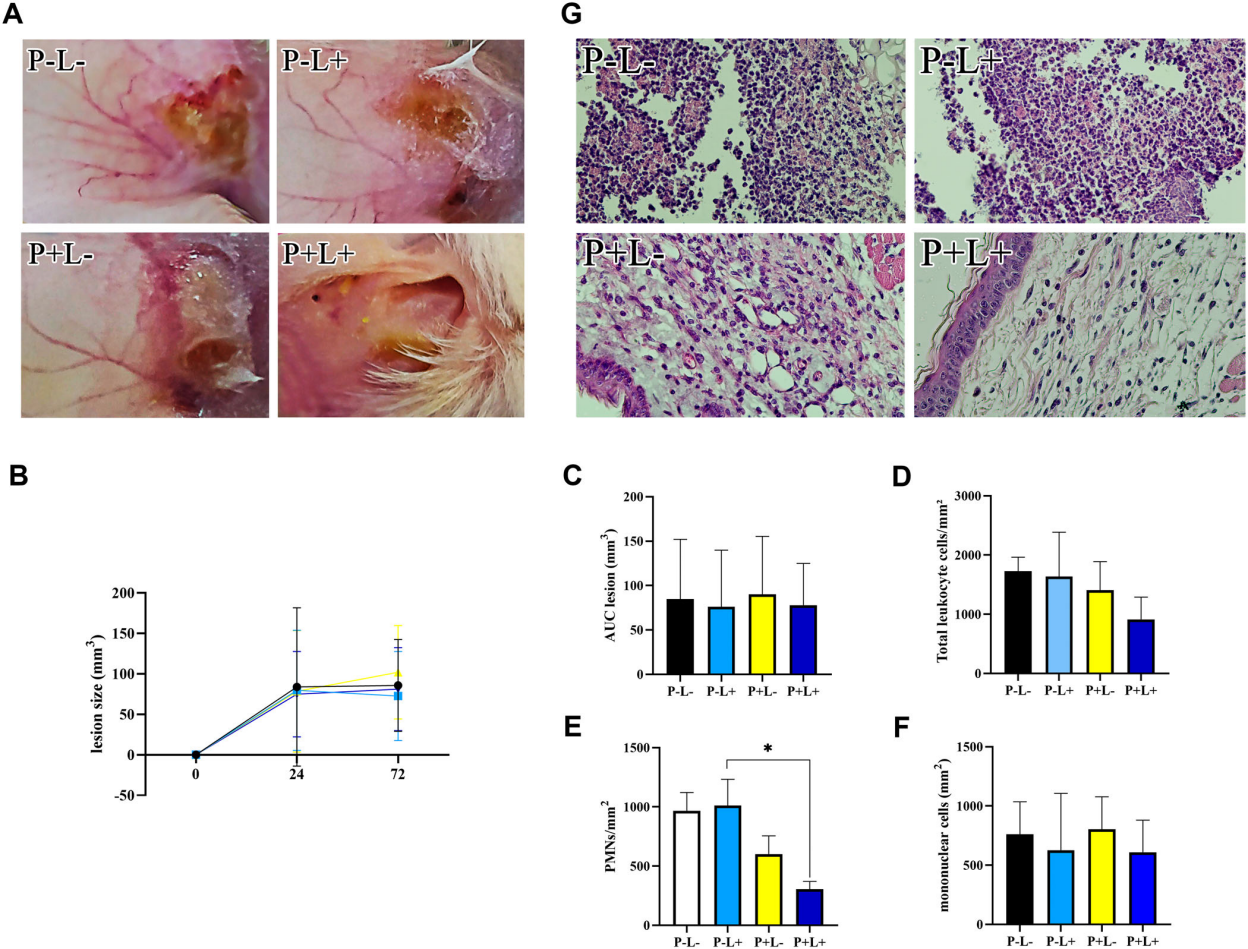
Inflammatory response at the infection site. (A) Macroscopic assessment of inflammation signs in the left ear 24 h posttreatment. (B) Lesion size measured and expressed in mm³. (C) AUC of lesion size over time. (D) Total leukocyte count in the inflammatory infiltrate. (E, F). Differential counts of polymorphonuclear and mononuclear cells. (G) Representative histological images for each experimental group. PMN, polymorphonuclear cells. *p < 0.05.

Macroscopic examination of the infection site revealed erythema, swelling, and abscess formation in all control groups, whereas tissue recovery was observed in the P+L+ treatment group (Figure 4A). No significant difference in lesion size was detected between groups (Figure 4B,C). Histological analysis of collected tissue samples was performed to assess total cellular influx (Figure 4D). A reduction in neutrophil number was observed in tissues from groups treated with quercetin and blue LED (Figure 4E).

### 3.6 Quercetin Increases the Strength of Interaction Between Cytokines in the Draining Lymph Node

**Figure 5:**
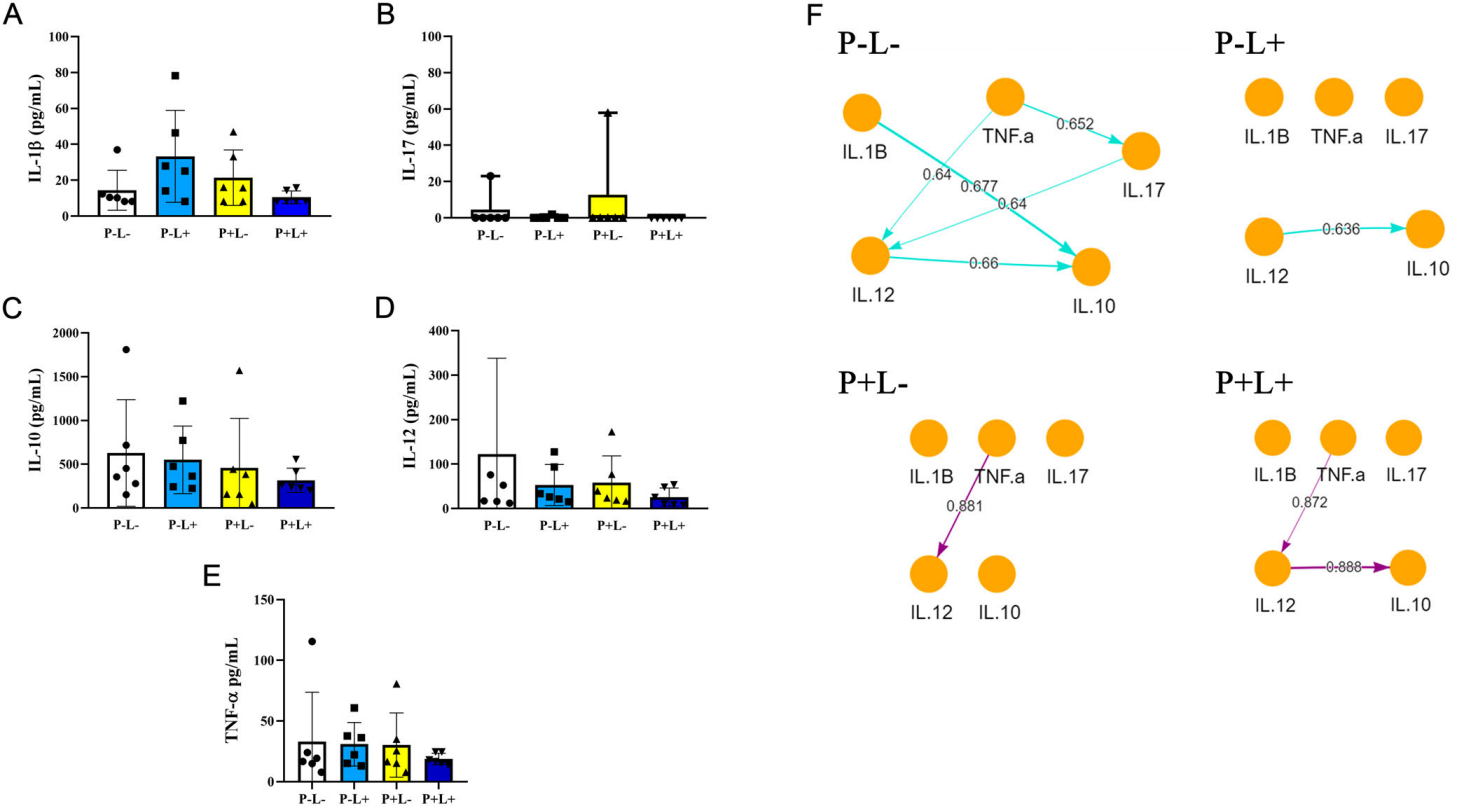
Quantifying cytokines in the draining lymph node (pg/mL). After 72 h of challenge with MRSA, the draining lymph nodes were removed, macerated in 1 mL of sterile saline solution, and analyzed by ELISA for the presence of IL‐1β (A), IL‐17A (B), IL‐12 (C), TNF‐α (D), and IL‐10 (E). Bayesian analysis (F) built with RStudio software showing the interactions among cytokines. Arrow direction indicates the causal relationship between two numerical variants, and the strength of the correlation is indicated by the colors of the arrows. The causal relationship is stronger as it approaches 1 and threshold = 0.8.

No significant differences in cytokine production were observed between the groups studied (Figure 5A–E). As we reported that there are no differences in the production of the cytokines studied, we sought to understand the relationship between them in the context studied. Through our data, we were able to visualize the interaction role that one cytokine plays over the other and how this interrelationship appears in the form of a network, modulate the pro‐inflammatory environment (Figure 5F). Using the Bayesian Network.boot.strength package, networks with interaction strength > 0.2 (threshold = 0.2) were generated, where the direction of the arrows shows the causal relationship between numerical variables. Our results show a strong interaction between TNF‐alpha and IL‐12 p70 (0.88) in the groups treated with quercetin (P+L−) and a greater interaction between IL‐12 p70 and IL‐10 (0.88) when we combined light and quercetin (P+L+).

## 4 Discussion

With the increasing resistance to conventional drugs, interest in alternative therapies has increased. In this context, PDT and the identification of novel PS have been the focus of extensive research. The present study investigates the use of quercetin as a photosensitizing agent for PDT in the treatment of an intradermal infection model caused by *S. aureus*. Quercetin has already been described in the literature as an antimicrobial compound against *S. aureus*. In this investigation, we demonstrate how the photodynamic activation of this isolated substance can promote bacterial clearance in vitro at lower doses than those described in the literature. Furthermore, the mechanism underlying its antimicrobial activity was evaluated, and we showed for the first time, through in vivo studies, the effectiveness of this antimicrobial action.

The extraction of compounds from plants is particularly relevant, as the development of an ideal PS requires some characteristics such as a high absorption coefficient within the excitation light spectrum, high quantum yield, and photostability (DeRosa 2002). Quercetin offers significant advantages in this context, being a plant‐derived flavonoid group with low toxicity (Figure 1A).

Among its various advantages presented, quercetin was selected due to its antimicrobial, antioxidant, and anti‐inflammatory properties (Hou et al. 2019; Nguyen and Bhattacharya 2022; Willian de Alencar Pereira et al. 2023). The literature reports the in vitro use of quercetin against methicillin‐resistant *S. aureus*, in synergism with other antibiotics or isolated at a MIC of 500 µg/mL (Amin et al. 2015; Júnior et al. 2018). However, in this study, we demonstrated that combining this substance with light significantly increases its antimicrobial efficacy. Following photoactivation protocol, the antimicrobial activity of quercetin increased (Figure 1B), enabling effective bacterial control at lower doses (100 µg/mL).

To elucidate the mechanism underlying the antimicrobial action of photoactivated quercetin, we performed the uric acid absorbance decay assay (Figure 2A). This assay has been previously described in the literature as a means to assess the rate of singlet oxygen production (dos Santos et al. 2019a). Our results indicate that quercetin's antimicrobial activity is mediated via singlet oxygen generation (Figure 2C), similar to the process observed with *M. cauliflora* and resveratrol (dos Santos et al. 2019a, 2019b). Moreover, the reaction occurs with remarkable speed, as shown in Figure 2A.

The zeta potential provides information on the potential interaction or bioadhesion of charged particles with the surfaces of microbial cells, allowing the evaluation of the mechanism that contribute to bacterial death (Lin et al. 2006; Fang et al. 2016; Raposo et al. 2023). In the presence of quercetin, the negative charge density of bacterial cell walls is altered, suggesting binding of the flavonoid surface to bacterial cell walls, corroborating findings reported in the literature for *E. coli* and *L. monocytogenes* (Lee et al. 2023), however, further studies are required to fully elucidate the mechanism of action.

*S. aureus* is a major pathogen of the integumentary system. For our in vivo study, we employed an intradermal infection model, widely used to replicate the inflammatory events observed in human *S. aureus* infections (Almeida et al. 2017; dos Santos et al. 2019a; Muniz et al. 2021). Contrary to reports in the literature regarding the anti‐inflammatory effect of light (Sadowska et al. 2021), the analysis of the lesion area showed no difference between the groups that received the incidence of blue LED light (Figure 4B), a fact explained by MRSA's potential to cause inflammation even after death.

In *S. aureus* infections, the host initiates an innate immune response primarily mediated by neutrophils (Rigby and DeLeo 2012), which migrate to the site of infection, phagocytizing the bacteria and migrating to the draining lymph nodes (Navarini et al. 2009; Miller and Cho 2011; Guggenberger et al. 2012; Tong et al. 2015). Therefore, histological analysis was performed to verify the type and number of cells at the site of infection. Previous studies have shown that PMN migration, when stimulated by LPS in vitro, was dose‐dependently reduced by quercetin treatment (Hwa et al. 2022). Consistent with these findings, we observed a reduction in the number of polymorphonuclear cells recruited to the inflammatory site in the groups treated with quercetin, corroborating the in vitro results (Figure 4E).

Adaptive immunity is activated when dendritic cells (DCs) migrate from infected tissues and present antigens to lymphocytes in draining lymph nodes that expand during active infection (Sokhi et al. 2022). As demonstrated with curcumin, resveratrol, and *M. cauliflora* (dos Santos et al. 2019a; Santos et al. 2019b; Muniz et al. 2021), the analysis of the bacterial load in the draining lymph node showed a difference between the P−L− (control group) and P+L+ (treated group) (Figure 3A), demonstrating the effectiveness of PDT in decreasing bacterial burden. In response to an infectious stimulus, draining lymph nodes undergo hyperplasia to increase the number of cells available to control the infection and organize an adequate defense against the pathogen. They secrete cytokines and chemokines that aid in recruiting neutrophils for an adequate immune response (Liao and von der Weid 2015).

Different states of DC maturation and activation can influence cytokine production of cytokines. Cytokines such as TNF‐α and IL‐12 are critical for DC activation and regulation of the cellular immune response. Literature reports indicate that the generation of cytokines (IL‐1α, IL‐1β, IL‐6, IL‐10 and IL‐12 p70) by activated DCs was impaired by quercetin treatment (Huang et al. 2010). Quercetin has anti‐inflammatory properties, with reduced secretion of IL‐18, TNF‐α, IL‐1β, and IL‐6 (Tang et al. 2019; Chiang et al. 2022). In our study, no significant differences in cytokine secretion were observed among the experimental groups (Figure 5A–E), unlike what was observed with other compounds (Almeida et al. 2017; dos Santos et al. 2019b). However, when we analyzed the relationship between cytokines, we observed a greater interaction between the cytokines TNF‐α and IL‐12p70, cytokines that stimulate phagocytic activity, in the groups with quercetin, which explains the greater bacterial clearance in these groups (Figure 5F).

The anti‐inflammatory properties of blue light have also been observed in previous studies. Fischer et al. (2013) reported that blue LED light reduced DC activation and decreased the secretion of IFN‐γ, IL‐2, IL‐10, IL‐12p70, IL‐1β, and TNF‐α by T cells, conferring an anti‐inflammatory effect (Fischer et al. 2013). Combining the known mild anti‐inflammatory action with the known anti‐inflammatory action of quercetin reinforces the benefit of new studies involving quercetin as PS in aPDT.

Despite the extensive literature demonstrating quercetin's anti‐inflammatory effects, more exploration of its impact on PDT in vivo models needs to be explored. Building upon our in vitro and in vivo data, we hypothesized that the photoactivation of quercetin reduces bacterial load at lower doses, thereby optimizing the treatment against *S. aureus*. This present study provides compelling evidence of an in vivo pathway exhibiting both anti‐inflammatory and antimicrobial effects. Furthermore, the antimicrobial activity demonstrated in this study reinforces the advantages of utilizing this flavonoid in aPDT, laying the groundwork for future investigations into the pathway involved in bacterial clearance through PDT, leveraging quercetin as a PS.

Our results reflect assessments carried out 72 h postinfection; further studies are warranted to evaluate the efficacy of quercetin‐based aPDT over longer time periods.

## Author Contributions

**Maria Poliana Leite Galantini:** conceptualization, validation, formal analysis, investigation, writing – original draft, writing – review and editing. **Caroline Vieira Gonçalves:** validation, methodology, investigation. **Amanda Kelle Santos Novaes:** validation, methodology, investigation. **Caio Oliveira Lopes de Magalhães:** validation, methodology, investigation. **Igor Pereira Ribeiro Muniz:** validation, methodology, investigation. **Israel Souza Ribeiro:** validation, methodology, investigation. **Paulo Henrique Bispo Lima:** validation, methodology, investigation. **Maria Elisa Santos Flores:** validation, methodology. **Artur Reis Carvalho:** validation, methodology**. Luísa Carregosa Santos:** validation, methodology, resources. **Daiana Silva Lopes:** conceptualization, methodology, formal analysis, data Curation. **Juliano Geraldo Amaral:** conceptualization, methodology, resources, supervision. **Robson Amaro Augusto da Silva:** term, conceptualization, data curation, methodology, resources, supervision, project administration, funding acquisition.

## Ethics Statement

The Committee for Ethics in the Use of Animals (CEUA) IMS‐CAT UFBA approved all animal use procedures under protocol number 112/2022.

## Conflicts of Interest

The authors declare no conflicts of interest.

## Data Availability

The data that support the findings of this study are available on request from the corresponding author. The data are not publicly available due to privacy or ethical restrictions.
